# RECRUITING FAMILY CAREGIVERS OF PERSONS LIVING WITH DEMENTIA FOR PARTICIPATION IN AN ONLINE INTERVENTION

**DOI:** 10.1093/geroni/igz038.2199

**Published:** 2019-11-08

**Authors:** Glenna Brewster, Fayron Epps, Rachel Nash, Patricia Griffiths, Janice Phillips, Joe Nocera, Raj Shah, Kenneth Hepburn

**Affiliations:** 1 Emory University Nell Hodgson Woodruff School of Nursing, Atlanta, Georgia, United States; 2 Georgia State University, Atlanta, Georgia, United States; 3 Emory University, Atlanta, Georgia, United States; 4 Atlanta VA Medical Center, Decatur, Georgia, United States; 5 Rush University, Chicago, Illinois, United States

## Abstract

Responsibilities of caregiving for persons living with dementia make it challenging to participate in in-person research studies. Caregivers may be more willing to participate in studies that are online. This presentation will highlight recruitment strategies of a 4-site telehealth caregiver intervention for caregivers of persons living with dementia. Thus far, we have recruited 596 participants over the period of 2 years: 76, 189, 164 and 167 from each of the sites, respectively. Community partnership strategies such as presentations at churches and events organized by the Alzheimer’s Association, and the Alzheimer’s Disease Research centers, using a handshake protocol, and using social media sites such as Facebook and Twitter have all been effective at recruiting participants. Ongoing communication among the staff at different sites is also an important aspect of successful recruitment. These strategies have enabled recruitment to continue at a consistent rate and enabled the maintenance of relationships within the community.

